# The Impact of Inflammation on Metabolomic Profiles in Patients With Arthritis

**DOI:** 10.1002/art.38021

**Published:** 2013-07-26

**Authors:** Stephen P Young, Sabrina R Kapoor, Mark R Viant, Jonathan J Byrne, Andrew Filer, Christopher D Buckley, George D Kitas, Karim Raza

**Affiliations:** 1 University of BirminghamBirmingham, UK; 2 University of Birmingham and the Sandwell and West Birmingham Hospitals NHS TrustBirmingham, UK; 3 University of Birmingham and the University Hospitals Birmingham NHS Foundation TrustBirmingham, UK; 4 University of Birmingham and the Dudley Group of Hospitals NHS Foundation TrustBirmingham, UK

## Abstract

***Objective.* Inflammatory arthritis is associated with systemic manifestations including alterations in metabolism. We used nuclear magnetic resonance (NMR) spectroscopy–based metabolomics to assess metabolic fingerprints in serum from patients with established rheumatoid arthritis (RA) and those with early arthritis.**

***Methods.* Serum samples were collected from newly presenting patients with established RA who were naive for disease-modifying antirheumatic drugs, matched healthy controls, and 2 groups of patients with synovitis of ≤3 months' duration whose outcomes were determined at clinical followup. Serum metabolomic profiles were assessed using 1-dimensional ^1^H-NMR spectroscopy. Discriminating metabolites were identified, and the relationships between metabolomic profiles and clinical variables including outcomes were examined.**

***Results.* The serum metabolic fingerprint in established RA was clearly distinct from that of healthy controls. In early arthritis, we were able to stratify the patients according to the level of current inflammation, with C-reactive protein correlating with metabolic differences in 2 separate groups (*P* < 0.001). Lactate and lipids were important discriminators of inflammatory burden in both early arthritis patient groups. The sensitivities and specificities of models to predict the development of either RA or persistent arthritis in patients with early arthritis were low.**

***Conclusion.* The metabolic fingerprint reflects inflammatory disease activity in patients with synovitis, demonstrating that underlying inflammatory processes drive significant changes in metabolism that can be measured in the peripheral blood. The identification of metabolic alterations may provide insights into disease mechanisms operating in patients with inflammatory arthritis.**

The etiology of rheumatoid arthritis (RA) is not fully understood but involves both genetic and environmental factors. In addition to synovitis, there are widespread systemic effects mediated by proinflammatory cytokines that impact on metabolism. Tumor necrosis factor α, interleukin-1 (IL-1), and IL-6 all promote cachexia, which is often associated with RA (1–2). The extent of the metabolic changes and the types of metabolites seen may therefore be good markers of cytokine-mediated inflammatory processes in RA. A novel systems approach to the assessment of metabolic changes in disease is metabolomics, which aims to investigate overall metabolic activity, and so takes into account the genetic and environmental background, allowing integration of the effects of these factors (3). Low serum levels of a number of specific metabolites, including histidine, have been reported in RA patients (4), and this metabolite discriminated osteoarthritis from RA (5). Lactate levels in synovial fluids also vary, with higher levels seen in seropositive, than in seronegative, RA (6). Changes in blood lipids in RA have been widely described (7–8). More recently, lipid changes (9) and alterations in a range of serum cytokines and chemokines (10) have been shown to predate the development of arthritis, suggesting that changes in metabolites might be observable early in the development of RA.

Altered metabolic fingerprints have been seen in a number of inflammatory diseases. For example, analysis of fecal extracts differentiated between normal controls, patients with Crohn's disease, and patients with ulcerative colitis. Significantly, these two patient groups could be distinguished using metabolic profiling (11), suggesting that the effects of inflammation on metabolism vary between conditions. This concept is supported by our previous work in which we used metabolic fingerprinting to differentiate between two otherwise similar inflammatory eye diseases (12) and between a number of neurologic conditions (13) and also to predict treatment responses in patients with inflammatory arthritis (14). With this background, we hypothesized that metabolomic profiles may be useful in predicting the development of RA in patients with early arthritis, an area where better predictive tools are currently needed (15). We also aimed to determine whether this analysis could provide novel insights into disease mechanisms in arthritis as has been the case in other conditions (16).

To investigate the potential of metabolic fingerprinting in inflammatory arthritis, we have applied a nuclear magnetic resonance (NMR)–based metabolomic approach to the analysis of serum from newly presenting patients with established RA and patients with early arthritis. We initially sought to assess whether the metabolic fingerprint in patients with established RA differed from that of healthy controls and then whether this fingerprint differed in patients with early arthritis in relation to the extent of inflammation and final outcomes.

## PATIENTS AND METHODS

**Patients.** Patients were recruited through the inflammatory arthritis clinic at Sandwell and West Birmingham Hospitals NHS Trust. The study was conducted in compliance with the Declaration of Helsinki, and ethical approval was obtained from the local ethics committee. All subjects gave written informed consent. Serum samples were collected from the patients and stored at −80°C until analyzed.

*Patients with established RA.* Serum samples were collected from 16 newly presenting patients who were naive for disease-modifying antirheumatic drugs (DMARDs), fulfilled the American College of Rheumatology (ACR) 1987 classification criteria for RA (17), and had a symptom duration of >3 months. Symptom onset was defined as the time of onset of inflammatory joint pain and/or early morning stiffness and/or joint-related soft tissue swelling.

*Patients with early arthritis.* Serum was collected from patients with early arthritis at the time of initial presentation. All patients had one or more swollen joints and symptoms (inflammatory joint pain, early morning stiffness, and/or joint-related soft tissue swelling) of ≤3 months' duration as previously described (18). Patients with evidence of previous inflammatory joint disease were excluded. Two groups of patients were studied, one recruited after the other. There were 89 patients with early arthritis in group 1 and 127 in group 2. Patients were followed up for 18 months and then assigned to their final diagnostic categories. Patients were classified as having RA according to the ACR 1987 criteria (17), allowing criteria to be satisfied cumulatively. Patients were diagnosed as having reactive arthritis, psoriatic arthritis, and a number of miscellaneous conditions according to established criteria. As previously described (18), patients were considered to have resolving arthritis if they had no evidence of joint-related soft tissue swelling on final examination and had not received DMARD or steroid treatment within the previous 3 months. Persistent disease was defined as persistent joint-related swelling or treatment with DMARDs or steroids for inflammatory joint symptoms (within the previous 3 months).

**Metabolomic analysis.** After thawing, serum samples were centrifuged at high speed (13,000*g*) and diluted 1:1 with an aqueous solution containing D_2_O (20%), NaCl (150 m*M*), trimethylsilyl 2,2,3,3-tetradeuteropropionic acid (TMSP) (1 m*M*), and sodium phosphate (20 m*M*) (pH 7). One-dimensional ^1^H spectra were acquired at 300°K using a standard spin-echo pulse sequence with water suppression using excitation sculpting on a Bruker DRX 500 MHz NMR spectrometer equipped with a cryoprobe. Spectra for patients with early arthritis in group 1 and group 2 were acquired several years apart and so were analyzed separately. Samples were processed and data were calibrated with respect to the TMSP signal. Spectra were read into ProMetab (19), custom written software in MatLab version 7 (The MathWorks), and were truncated to the chemical shift range of 0.8–10.0 parts per million. Spectra were segmented into 0.005-ppm (2.5 Hz) chemical shift “bins” and the spectral area within each bin was integrated. Spectra were corrected for baseline offset and then normalized to a total spectral area of unity, and a generalized log transformation was applied (19–20). Binned data were then compiled into a matrix, with each row representing an individual sample.

**Statistical analysis.** Data bins were mean centered and subjected to principal components analysis (PCA) using the PLS_Toolbox (version 5.8) (Eigenvector Research) in MatLab (release 2009a; MathWorks). Partial least-squares discriminant analysis (PLS-DA) was used to perform supervised clustering of samples (21–22) and on some occasions an orthogonal signal correction was applied (OPLS-DA) to enhance the separation of groups (23). The PLS-DA models were cross-validated using Venetian blinds (21–22), a method which reassigns randomly selected blocks of data to the PLS-DA model to determine the accuracy of the model in correctly assigning class membership. The application of such methods to clinical studies has been demonstrated in many studies and has recently been reviewed by Nicholson et al (24). For all of the PLS-DA analyses, the variances of Y-block explained by individual latent variables (LVs) are available from the corresponding author upon request.

Partial least-squares regression analysis (PLS-R), a regression method that identifies which metabolites can predict a continuous variable, was also used to investigate the relationship between the metabolic fingerprint and inflammatory burden, with C-reactive protein (CRP) being used as the dependent variable. This analysis yields R^2^, a measure of the cross-validated goodness-of-fit of the linear regression, while permutation testing (multiple analyses using random data subsets) was used to assess the significance of this prediction.

Lists of metabolites providing the greatest discrimination between groups were then identified for each technique. A diagram demonstrating the methodologies used is shown in Supplementary Figure 1, available on the *Arthritis & Rheumatism* web site at http://onlinelibrary.wiley.com/doi/10.1002/art.38021/abstract. Using multivariate analyses, peaks with large loadings (for PCA), weightings (for PLS-DA), or regression coefficients (for PLS-R) were identified. NMR databases (Human Metabolome Database version 2.5 #x005B;25#x005D; and Chenomx NMR suite; Chenomx, professional version 4.0 #x005B;26#x005D;) were used to identify the metabolites. Published data on metabolites identified in human sera were also used to guide identification (27–28).

**Figure 1:**
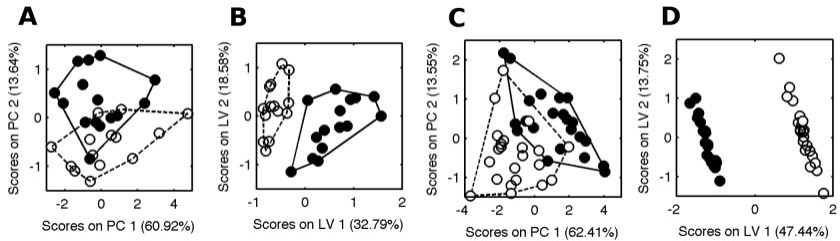
Metabolic fingerprinting distinguishes between sera from patients with established rheumatoid arthritis (RA) and matched healthy controls and illustrates that metabolomic profiles in patients with early arthritis are altered by control of or resolution of inflammation. A and B, One-dimensional ^1^H–nuclear magnetic resonance (NMR) spectra of serum obtained from disease-modifying antirheumatic drug–naive patients with RA (solid circles) and healthy controls (open circles) were subjected to principal components analysis (PCA) (A) and to supervised analysis (partial least-squares discriminant analysis) (B). C and D One-dimensional ^1^H-NMR spectra of serum obtained from a subset of patients with early arthritis before (solid circles) and after (open circles) a decrease in the C-reactive protein level following therapy or spontaneous resolution were subjected to PCA (C) and to supervised analysis (partial least-squares discriminant analysis with orthogonal signal correction) (D). The values on the axis labels indicate the proportion of the variance captured by each principal component (PC1 and PC2) or latent variable (LV1 and LV2).

## RESULTS

### Different metabolomic profiles in patients with established RA compared to healthy controls

The characteristics of the patients with established RA and age- and sex-matched healthy controls are shown in Table1. The median Disease Activity Score in 28 joints using the erythrocyte sedimentation rate in the RA patients was 5.88 (interquartile range #x005B;IQR#x005D; 5.25–6.99). PCA was used to generate an unbiased overview of the major metabolic differences between patients with established RA and control individuals. There was separation of the 2 groups that was largely dependent on principal component 2 (Figure 1A), the scores of which were significantly higher in the RA group (*P* < 0.0001 by *t*-test). To help discover the major discriminatory metabolites, these data were subjected to supervised analysis using OPLS-DA. Discriminatory metabolites are shown in Table2 (column 1).

**Table 1:** Baseline characteristics of the patients with established RA, healthy controls, and patients with early arthritis*

	Patients with established RA (n = 16)	Healthy controls (n = 14)	Patients with early arthritis in group 1 (n = 89)	Patients with early arthritis in group 2 (n = 127)	*P*†
Age, median (IQR) years	57 (37–79)	54 (40–72)	46 (36–61)	50 (35–65)	0.32‡
Sex, no. (%) female	12 (75)	9 (64)	49 (55)	69 (54)	1.0§
Symptom duration, median (IQR) weeks	31 (18–52)	–	5 (2–9)	6 (4–9)	0.02‡
No. (%) taking NSAIDs	7 (44)	0 (0)	58 (65)	69 (54)	0.12§
CRP, median (IQR) mg/ml¶	20.5 (7.5–55.5)	–	19 (5.5–54)	19.5 (5.25–44.75)	0.79‡
RF positive, no. (%)	12 (75)	–	12 (13)	30 (24)	0.83§
Anti-CCP antibody positive, no. (%)#	9 (56)	–	13 (15)	29 (23)	0.39§
No. (%) with persistent arthritis	–	–	33 (37)	87 (68)	<0.0001‡
No. (%) with persistent arthritis who developed RA	–	–	18 (54)	55 (63)	0.41‡

* RA = rheumatoid arthritis; IQR = interquartile range; NSAIDs = nonsteroidal antiinflammatory drugs; RF = rheumatoid factor.

† Patients with early arthritis in group 1 versus patients with early arthritis in group 2.

‡ By Mann-Whitney test.

§ By Fisher's exact test.

¶ Data on C-reactive protein (CRP) were available for 84 patients in group 1 and 126 patients in group 2.

# Data on anti–cyclic citrullinated peptide (anti-CCP) were available for 126 patients in group 2.

**Table 2:** Metabolites contributing to the differentiation between groups, determined by analysis of PLS-DA weightings*

Metabolite, ppm	RA patients versus controls	Patients with early arthritis before versus after resolution of inflammation	Patients with persistent arthritis versus patients with resolving arthritis (group 1)	Patients with persistent arthritis versus patients with resolving arthritis (group 2)	Patients with persistent RA versus patients with resolving arthritis (group 1)	Patients with persistent RA versus patients with resolving arthritis (group 2)
LDL-CH3, 0.80	Low (6.30)	Low (3.03)	Low (6.81)	Low (2.87)	–	–
LDL-CH2, 1.21	Low (7.06)	Low (31.81)	Low (7.40)	Low (6.89)	–	Low (1.58)
3-hydroxybutyrate, 1.18, 1.19	High (4.21)	High (7.90)	–	High (6.87)	–	–
Lactate, 1.31, 4.11	High (54.51)	–	Low (12.85)	High (27.90)	Low (12.74)	High (16.98)
Alanine, 1.46, 1.48	Low (20.00)	Low (2.15)	–	–	–	Low (3.84)
Acetylglycine, 2.03	High (48.67)	High (17.41)	High (6.55)	High (6.80)	High (4.57)	Low (1.94)
Methylguanidine, 2.81	Low (10.17)	–	High (92.72)	Low (38.15)	High (34.76)	Low (6.51)
Taurine, 3.26	High (8.12)	High (9.11)	–	High (15.73)	–	High (8.66)
Glucose, 3.25, 3.88	High (16.8)	High (12.72)	–	High (11.55)	–	High (7.49)
Lipid, 5.32	Low (2.36)	Low (2.53)	–	–	–	–
Urea, 5.79	–	High (1.32)	High (3.90)	–	High (1.25)	–

* “High” indicates that the metabolite was at a higher concentration in the rheumatoid arthritis (RA; column 2), early arthritis before resolution (column 3), persistent arthritis (columns 4 and 5), or persistent RA (columns 6 and 7) phenotypes. Nuclear magnetic resonance chemical shifts (in parts per million), which identify the location of the major peaks in the spectra, are shown for each metabolite. Values in parentheses are the variable importance of the projection for each metabolite. PLS-DA = partial least-squares discriminant analysis; LDL-CH3 = low-density lipoprotein CH3.

Supervised analysis enhanced the separation of the 2 groups, and the optimized model comprised 4 LVs that captured 97.8% of the variance in the metabolic data. The major contributions to the separation were made by LV1 and LV2 (Figure 1B). This model was cross-validated using the Venetian blinds approach and identified samples from the RA patients with a sensitivity of 93.3% and a specificity of 100%.

### Resolution of inflammation is reflected in metabolomic profile changes

In 22 patients with early arthritis and active disease at baseline (median CRP 66 #x005B;IQR 47–131#x005D;), serum samples were available at a followup time point (a median of 49 weeks #x005B;IQR 24–69#x005D; from the time of initial assessment and sample collection) at which CRP levels were significantly lower (median <5 #x005B;IQR <5 to <5#x005D;). This decrease in CRP was associated with either spontaneous resolution of disease or control of disease with therapy. Unbiased PCA discriminated between samples at baseline and followup (Figure 1C), and OPLS-DA produced a model comprising 2 LVs that discriminated between these samples with 100% specificity and sensitivity (Figure 1D), demonstrating the influence of inflammation on the metabolic fingerprint.

To provide an overall description of which regions discriminated between groups in the PLS-DA analyses, weightings plots derived from the models were generated. Some common features were seen in these weightings plots from PLS-DA models separating RA patients from controls and in the analysis of the patients with early synovitis from whom a second serum sample had been collected at a time when the CRP had decreased significantly (Table2 column 2).

### Association of metabolomic profiles with inflammation in patients with early arthritis

The characteristics of the 89 patients with early arthritis in group 1 and the 127 patients with early arthritis in group 2 are shown in Table1.

**Figure 2:**
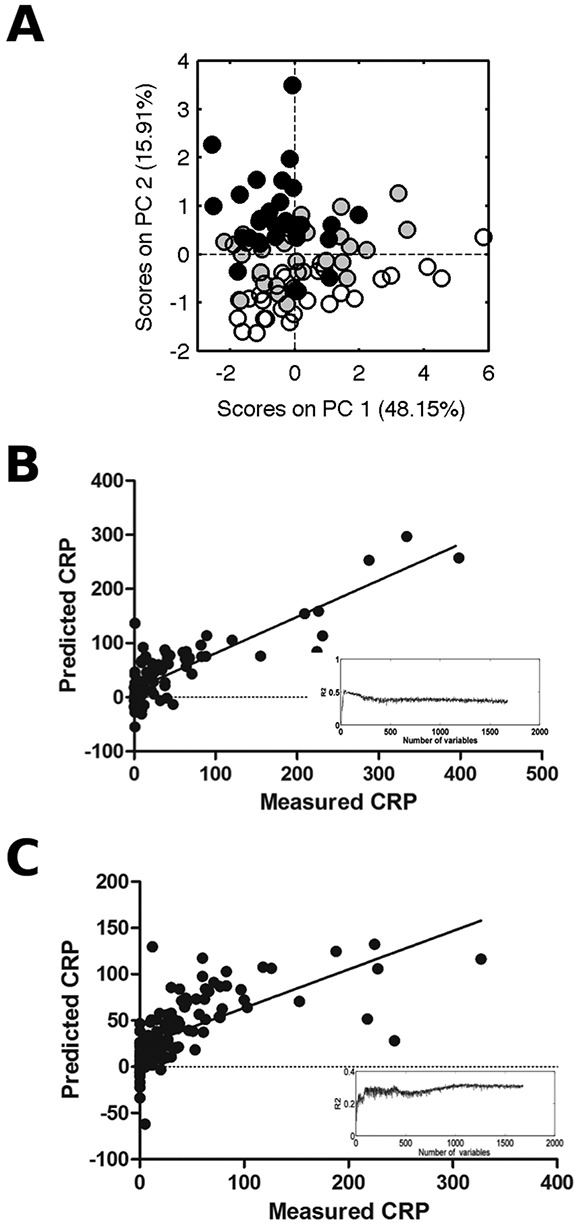
The metabolic fingerprints of sera from patients with early arthritis prior to treatment with disease-modifying antirheumatic drugs are strongly influenced by the level of inflammation. A, One-dimensional ^1^H-NMR spectra of serum obtained from patients with very early arthritis (in group 1) were subjected to PCA. Solid circles indicate C-reactive protein (CRP) levels in the highest tertile, shaded circles indicate CRP levels in the middle tertile, and open circles indicate CRP levels in the lowest tertile. B and C, Strong correlations between measured CRP and predicted CRP values were found for patients with early arthritis in group 1 (B) and those in group 2 (C) (*P* < 0.001 for both groups). The predicted values were calculated from the concentrations of a series of metabolites that were discovered using partial least-squares regression analysis. Insets show the optimization of the multivariate regression, with the highest correlation between measured and predicted CRP occurring with 154 NMR bins (maximum R^2^ of 0.671) for group 1 and with 1,136 NMR bins (maximum R^2^ of 0.4157) for group 2. See Figure 1 for other definitions.

PCA of NMR spectra of serum from 84 patients with early arthritis (in group 1) in whom the CRP levels were known showed a broad spread on the scores plot (Figure 2A). When the CRP level for each sample was superimposed on the PCA plot, it was clear that the level of inflammation at the time of sampling influenced the distribution. To investigate this further, the relationship between baseline metabolomic profiles and CRP was assessed using PLS-R. The analysis used a forward selection approach to discover those NMR bins that were most predictive. A total of 154 bins created the optimal model with a cross-validated R^2^ of 0.671 (Figure 2B). Permutation testing with these 154 NMR bins demonstrated that the regression model was statistically valid (*P* < 0.001).

To further validate this relationship between the level of inflammation and baseline metabolomic profiles, the PLS-R analysis was carried out in a separate group of 127 patients with early arthritis (group 2). The maximum R^2^ for the regression of the real (i.e., ordered) data was 0.416 (Figure 2C). Permutation testing with 1,136 NMR bins included (as optimized by forward selection) again demonstrated that the regression model was statistically valid (*P* < 0.001).

It is known that metabolic status and products of metabolism are influenced by several variables, including age, sex, hypertension, diabetes mellitus, hyperuricemia, and smoking, but when we adjusted our analysis for these confounding effects our results remained significant (data are available from the corresponding author upon request). We also excluded the effect of medication used at the time of sample collection (steroids, DMARDs, and nonsteroidal antiinflammatory drugs #x005B;NSAIDs#x005D;) and found that the relationship between CRP and metabolomic profile was still significant (data are available from the corresponding author upon request).

The regression analysis was carried out to examine the relationship between metabolomic profiles and CRP without the confounding effect of variables that are known to influence metabolic status by assessing only individuals who were not current smokers (n = 67 in group 1 and n = 96 in group 2) and individuals without hypertension (n = 70 in group 1 and n = 96 in group 2), diabetes mellitus (n = 89 in group 1 and n = 118 in group 2), or hyperuricemia (defined as a uric acid level of >340 μmoles/liter; n = 58 in group 1 and n = 67 in group 2). The effects of DMARDs and steroids on metabolic status were also excluded by carrying out the regression analyses only for those patients who were not taking DMARDs (n = 85 in group 1 and n = 125 in group 2; the few patients who were taking DMARDs were taking them for other comorbidities, e.g., inflammatory bowel disease) or steroids (n = 86 in group 1 and n = 123 in group 2) at the time of recruitment into the study. We excluded the effect of NSAIDs by only carrying out the regression analysis for those patients who were taking NSAIDs at time of recruitment since the majority of patients were taking NSAIDs (n = 60 in group 1 and n = 66 in group 2). The relationship between the CRP level and metabolomic profile was statistically significant for all analyses, except for when women alone in group 1 were analyzed (data are available from the corresponding author upon request).

We also used the PLS-R analysis that related the metabolic fingerprint to CRP level to generate a rank order of NMR bins that identified the regions of the spectra that most strongly predicted the inflammatory burden in groups 1 and 2 (Table3). Almost identical groups of metabolites were identified for both patient groups, suggesting a complex but real relationship between metabolism and inflammatory burden.

**Table 3:** Metabolites most strongly correlated with CRP level in patients with early arthritis in groups 1 and 2*

Ranked importance	Metabolites identified in patient group 1 (ppm)	Metabolites identified in patient group 2 (ppm)
1	Choline (3.20, 3.22, 3.23)	LDL lipids (1.24–1.27)
2	LDL lipids (1.24–1.27)	Acetylglycine (2.03, 3.71, 3.76)
3	Lactate (1.31, 1.33, 4.11)	Glucose (3.24–3.26, 3.41, 3.48, 3.68–3.69, 3.88)
4	Acetylglycine (2.03, 3.71, 3.76)	Fatty acids (0.8–0.84, 2.22–2.24)
5	Urea (5.77, 5.78, 5.79, 5.80, 5.81, 5.82)	Methylguanidine (2.81)
6	Glucose (3.24–3.26, 3.41, 3.48, 3.68–3.69, 3.88)	Lactate (1.31, 1.33)
7	Methylguanidine (2.81)	Threonine (3.58)
8	Methylhistidine (3.70)	Homocysteine (3.86)
9	Cholesterol (0.91)	Glycine (3.55)
10	Taurine (3.42)	Taurine (3.42)
11	Threonine (3.58)	Methylxanthine (3.49)
12	Fatty acids (0.8–0.84, 2.22–2.24)	Choline (3.20, 3.22, 3.23)
13	Methylxanthine (3.49)	Methylhistidine (3.70)
14	Homocysteine (3.86)	Cholesterol (0.91)

* Metabolites were identified using the partial least-squares regression analysis model and represent the regions of the spectra which had the greatest influence on the correlation with C-reactive protein (CRP) level. Values in parentheses are the nuclear magnetic resonance chemical shifts (in parts per million), which identify the location of the major peaks in the spectra. LDL = low-density lipoprotein.

### Relationships between clinical outcomes and metabolomic profiles in patients with early arthritis

**Figure 3:**
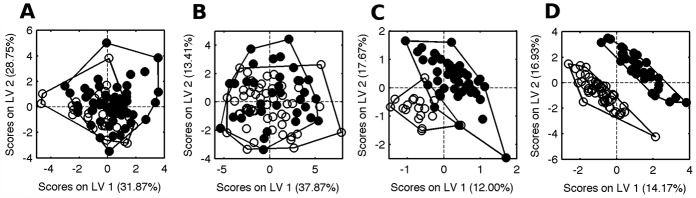
Metabolic fingerprints of sera from patients with early arthritis from 2 different patient groups. A and B, Serum samples from patients with early arthritis from group 1 (A) and group 2 (B) at first presentation were assessed using partial least-squares discriminant analysis to distinguish patients whose disease was resolving (solid circles) from those whose disease was persistent (open circles). Sensitivity and specificity were 59.4% and 58.9%, respectively, for group 1 and 59.5% and 56.4%, respectively, for group 2. C and D, Serum samples from patients with early arthritis from group 1 (C) and group 2 (D) at first presentation were assessed using partial least-squares discriminant analysis with orthogonal signal correction, to distinguish patients whose disease was resolving (solid circles) from those who developed persistent RA (open circles). Sensitivity and specificity were 50% and 69.6%, respectively, for group 1 and 73.1% and 67.6%, respectively, for group 2. See Figure 1 for definitions.

A PLS-DA model was used to attempt to separate patients with an outcome of resolving arthritis from those with an outcome of persistent arthritis for both patient groups 1 and 2. The model separated the patients with regard to outcome but did so with only moderate sensitivity and specificity, of 59.4% and 58.9%, respectively, for group 1 using 5 LVs (Figure 3A) and 59.5% and 56.4%, respectively, for group 2 using 6 LVs (Figure 3B). There was also no differentiation between patients with resolving disease and those with persistent RA in group 1 (Figure 3C) (sensitivity of 50% and specificity of 69.6% using 2 LVs), though there was good separation for group 2 (Figure 3D) with a sensitivity of 73.1% and specificity of 67.6% using 3 LVs.

There was no differentiation between patients with RA and non-RA outcomes for either cohort (see Supplementary Figure 2, available on the *Arthritis & Rheumatism* web site at http://onlinelibrary.wiley.com/doi/10.1002/art.38021/abstract). The variances of Y-block explained by individual LVs are available from the corresponding author upon request. Previous data have shown that treatment can affect metabolomic profiles (29), but there was no significant difference between the groups with regard to the number of patients taking NSAIDs (Table1), and there was no significant difference in the use of NSAIDs between patients with persistent disease and those with resolving disease for group 1 (*P* = 0.13) or group 2 (*P* = 0.1) or between patients with persistent RA and those with resolving disease for group 1 (*P* = 0.18) or group 2 (*P* = 0.17).

Metabolites were identified that contributed the greatest to the separation between patients with early arthritis with resolving disease and those with persistent disease, and also between patients with resolving disease and those with persistent RA for both patient groups (Table2). Although some predictive metabolites were common to both patient groups, there were some key differences in metabolites between the groups, suggesting that metabolomic techniques as applied in this study cannot fully discriminate between resolving and persistent outcomes or resolving disease and persistent RA in patients with early arthritis.

## DISCUSSION

Our data demonstrate that the serum metabolic fingerprint of patients with active established RA differs from that of healthy controls and that the serum metabolic fingerprint of patients with early arthritis varies depending on the level of inflammation. These results from patients with established RA are broadly consistent with a recent study using a similar experimental approach that focused on responses to therapy in a cohort of RA patients (30). There is also some overlap in discriminating metabolites identified in a recent mass spectrometry–based analysis of established RA (31). However, patients in both of those cohorts had already been exposed to DMARD therapy, and so it is possible that the therapy might have influenced their metabolic fingerprints. Our cohort of patients was sampled before any DMARD therapy and so was not exposed to this confounding factor.

Consistent with the findings of Lauridsen et al (30), we found that decreased NMR lipid signals were particularly discriminatory between different groups. Madsen et al (31), using mass spectrometry, observed changes in cholesterol. Such lipid changes were not seen in a mouse model of arthritis (32), probably because the investigators filtered samples to remove protein and the associated lipids. Changes in lipid profiles in the blood of RA patients have been widely described, and have been suggested to be a major contributing factor to the accelerated atherosclerosis associated with RA (7–8).

We found that 3-hydroxybutyrate was elevated in the patients with established RA compared to controls. This metabolite is known to be present in RA synovial fluid (33), in pouch fluid from the rat air-pouch model (34), and in the blood of mice with experimental arthritis (32). The presence of 3-hydroxybutyrate, a ketone body, suggests an increased level of lipolysis in RA patients compared with controls. This may be another explanation for the decreased levels of lipid that we have observed, and is consistent with the findings of earlier spectroscopic studies of synovial fluid (33), which determined that lipid metabolism may be a predominant source of energy in the hypoxic inflammatory joint.

Our PLS-R analysis revealed a clear correlation between the CRP level and the metabolites present. This was replicated in a separate group of patients. This approach indicated that increased concentrations of many of the same metabolites discovered in the PCA and OPLS-DA models, for example, low-density lipoprotein lipids, lactate, glucose, taurine, acetylglycine, and methylguanidine, were predictive of the inflammatory phenotype. However, a number of amino acids (e.g., choline, threonine, and methylhistidine) were also found to contribute strongly to the correlation (Table3). Thirteen of the 14 metabolites that were most strongly associated with CRP were the same for both groups of patients.

Many of the metabolites that were correlated with inflammation, as measured by serum CRP levels, are associated with lipid metabolism and may contribute to the increased levels of atherosclerosis associated with inflammatory disease. Abnormalities in lipids have been associated with pre-RA (35), early RA (36–37), and established RA (38,39), and lipid levels correlate with CRP even in the absence of clinically apparent inflammatory disease (41). Furthermore, lipid-lowering therapies influence inflammation (42–43) and antiinflammatory therapies influence lipids (44), demonstrating a complex relationship between inflammation and lipid metabolites which our data help to dissect.

Blood donors who went on to develop RA at least a decade later have been shown to have significantly more atherogenic blood lipid profiles than those who did not develop RA (45). While this difference did not correlate with CRP levels (45), it does suggest that lipid profiles early in disease might be predictive of outcome. However, in the present study we were unable to discriminate between patients with early arthritis who went on to develop persistent disease and those whose arthritis resolved. Although there was some separation between patients with persistent disease and those with resolving outcomes in both groups, the discriminating metabolites differed. This may result from the heterogeneity between the 2 groups of patients with early arthritis, for example, in terms of the proportion whose disease persisted (Table1), and so to confirm and extend our observations, these analyses need to be replicated in independent cohorts.

In conclusion, the metabolic fingerprint reflects inflammatory disease activity in patients with new-onset arthritis. This suggests that the underlying inflammatory processes drive significant changes in metabolism that can be measured in the peripheral blood. This may give us further insights into the mechanism of inflammation in inflammatory arthritis, as has been the case following the identification of contributing metabolites in other diseases (16). Furthermore, metabolomics may prove useful as a measure of the extent of disease, potentially separating patients with low disease activity states from patients in true remission.

## AUTHOR CONTRIBUTIONS

All authors were involved in drafting the article or revising it critically for important intellectual content, and all authors approved the final version to be published. Dr. Young had full access to all of the data in the study and takes responsibility for the integrity of the data and the accuracy of the data analysis.

**Study conception and design.** Young, Kapoor, Filer, Buckley, Raza.

**Acquisition of data.** Young, Kapoor, Filer.

**Analysis and interpretation of data.** Young, Kapoor, Viant, Byrne. Kitas, Raza.
