# Supplementary material for: The Impact of Inflammation on Metabolomic Profiles in Patients With Arthritis
Source: Arthritis Rheum. 2013 Jul 26;65(8):2015–23. doi: 10.1002/art.38021 (PMC3840700; doi:10.1002/art.38021)
Supplement: Supplementary file 1 [file art0065-2015-sd1.docx]

**Supplementary figure 1** Diagramatic illustration of the methodologies involved in the analysis of metabolomic data acquired using NMR based techniques. **A** represents an NMR spectrum obtained from a biological fluid – in this case serum. The x axis shows the chemical shift in parts per million (ppm) and the position on this axis is characteristic of particular chemical groups within each metabolite. The y axis shows the intensity of the peak signals. The spectrum is then segmented into bins (areas of fixed distance) to obtain figure **B.** The data bins from groups of spectra are then assessed by principal component analysis (PCA; figure **C**) or by a supervised analytical approach (Partial Least Squares Discrimant Analysis (PLSDA); figure **D**). The position on the axes of the PCA and PLSDA plots shows the extent to which the major features of the group of spectra are represented in each sample. The % of the covariance within the dataset which is captured by each component plotted is also shown on the axis. PCA, by identifying major features in the set of spectra, allows all the data from an entire spectrum to be condensed into a single dot **(C).** PCA identifies regions of the spectrum which allow the segregation and a loadings plot **(E)** can be used to identify the metabolites responsible for this segregation. PLSDA builds a model to give maximum discrimination between groups of samples **(D)** but is cross validated using a “venetian blinds” approach in which multiple versions of the model are generated after systematically excluding groups of samples. This allows a sensitivity and specificity for the model to be obtained. PLSDA also identifies regions of the spectrum which allow maximum segregation between pre-identified groups and a loadings plot **(E)** can be used to identify the metabolites responsible for this segregation.
