# Supplementary figures and images for: The Impact of Inflammation on Metabolomic Profiles in Patients With Arthritis
Source: Arthritis Rheum. 2013 Jul 26;65(8):2015–23. doi: 10.1002/art.38021 (PMC3840700; doi:10.1002/art.38021)

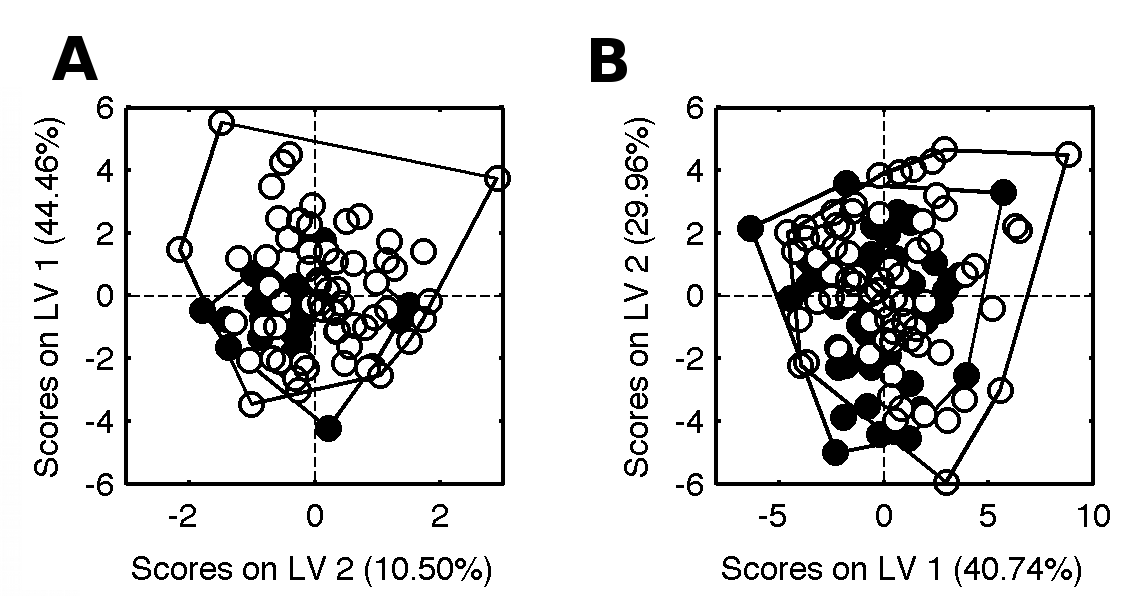

Supplement: Supplementary file 2 [file art0065-2015-sd2.tif]
